# Integration and Visualization of Translational Medicine Data for Better Understanding of Human Diseases

**DOI:** 10.1089/big.2015.0057

**Published:** 2016-06-01

**Authors:** Venkata Satagopam, Wei Gu, Serge Eifes, Piotr Gawron, Marek Ostaszewski, Stephan Gebel, Adriano Barbosa-Silva, Rudi Balling, Reinhard Schneider

**Affiliations:** ^1^ Luxembourg Centre for Systems Biomedicine, University of Luxembourg, Esch-Belval, Luxembourg.; ^2^ Information Technology for Translational Medicine (ITTM) S.A., Esch-Belval, Luxembourg.

**Keywords:** big data analytics, big data infrastructure design, data acquisition and cleaning, data integration, data mining, disease map

## Abstract

Translational medicine is a domain turning results of basic life science research into new tools and methods in a clinical environment, for example, as new diagnostics or therapies. Nowadays, the process of translation is supported by large amounts of heterogeneous data ranging from medical data to a whole range of -omics data. It is not only a great opportunity but also a great challenge, as translational medicine big data is difficult to integrate and analyze, and requires the involvement of biomedical experts for the data processing. We show here that visualization and interoperable workflows, combining multiple complex steps, can address at least parts of the challenge. In this article, we present an integrated workflow for exploring, analysis, and interpretation of translational medicine data in the context of human health. Three Web services—tranSMART, a Galaxy Server, and a MINERVA platform—are combined into one big data pipeline. Native visualization capabilities enable the biomedical experts to get a comprehensive overview and control over separate steps of the workflow. The capabilities of tranSMART enable a flexible filtering of multidimensional integrated data sets to create subsets suitable for downstream processing. A Galaxy Server offers visually aided construction of analytical pipelines, with the use of existing or custom components. A MINERVA platform supports the exploration of health and disease-related mechanisms in a contextualized analytical visualization system. We demonstrate the utility of our workflow by illustrating its subsequent steps using an existing data set, for which we propose a filtering scheme, an analytical pipeline, and a corresponding visualization of analytical results. The workflow is available as a sandbox environment, where readers can work with the described setup themselves. Overall, our work shows how visualization and interfacing of big data processing services facilitate exploration, analysis, and interpretation of translational medicine data.

## Introduction

Translational medicine capitalizes on advances in basic life sciences to improve clinical research and care. We witness great technological advances in methods characterizing human health and disease, including genetic and environmental factors of our well-being. This is a great opportunity to understand diseases and to find new diagnoses and treatments. However, the progress comes at a cost—translational research data sets nowadays include genomic, imaging, and clinical data sources,^1,2^ making them large and heterogeneous. In effect, important steps of the data life cycle in discovery—integration, analysis, and interpretation—are a challenge for biomedical research. Moreover, enabling biomedical experts to efficiently use big data processing pipelines is another challenge.

As translational medicine data become more and more rich and complex, their potential in informing both clinical and basic research grows.^3^ With constantly increasing presence of high-throughput molecular profiling, it becomes increasingly important to ensure that data interpretation capabilities follow generation of large-scale biomedical data sets.^4,5^ Visualization can support greatly the processing of complex data sets on each of the steps of the data life cycle. This opportunity is actively explored in various domains of biomedical research, including clinical big data^6^ or multiscale biomedical ontologies.^7^

Modern translational medicine approaches aim to combine clinical and molecular profiles of the patients to formulate informed hypothesis on the basis of stratified data.^8^ Integration of plethora of sources renders these data sets complex and difficult to process. Visualization of such integrated data sets can aid exploration and selection of key dimensions and subsets for downstream analysis. In turn, visually aided data analysis allows to comprehend even complicated workflows and aids interpretation of resulting data.

In this article, we demonstrate a workflow for translational medicine big data, in which visualization is an important component at each step of data processing and exploration. We describe in detail the interfaces allowing the construction of the workflow, followed up by a use case scenario. We conclude with a discussion of the results and an outlook for future development of visualization in biomedical big data exploration.

## Related Work

### Clinical and molecular (omics) data integration platforms

The rise of personalized medicine and the availability of high-throughput molecular analysis drives the development of storage, analytics, and interpretive methods to enable the transformation of increasingly voluminous biomedical data into proactive, predictive, preventative, and participatory healthcare.^3,9,10^ Key properties of biomedical big data in translational medicine, according to the “5V” classification,^11^ besides its volume are variety and veracity. A combination of clinical* and high-throughput molecular profiles (“omics”)^†^ creates a very variable heterogeneous data set, where dimensionalities of different data types span several orders of magnitude.^12^ Moreover, ensuring veracity, that is, quality, to clinical data is a challenging and time-consuming task.^13,14^ This stems from a variety of collection methods, featuring manual data input, nondigital data capture, and nonstandard formats. It needs to be stressed that proper data curation is a mandatory step for accurate analysis of clinical data and proper interpretation of analytical results.

The emergence of big biomedical data sets, covering dozens of thousands of patients,^12^ raises questions on infrastructure necessary to host and analyze them. Especially genomic data, generated rapidly due to dropping sequencing costs, pose a problem in terms of storage and analytics. The perspective of cloud computing is postulated as a solution to this challenge, as summarized in recent and extensive reviews.^5,15,16^ Nevertheless, due to ethical and legal issues arising in cloud-based scenarios,^17^ incorporation of clinical data and processing of sensitive omics are still considered an open question.

Translational medicine platforms integrating clinical and omics data need to ensure a protected environment for sensitive data processing. A number of solutions were developed to address this challenge, as summarized in an excellent review by Canuel et al.^18^ Platforms integrating clinical and omics data can be divided into two groups: repositories with an existing infrastructure and solutions requiring deployment. The first group is represented by technologies, such as STRIDE,^19^ iDASH,^20^ caGRID, and its follow-up, TRIAD^21,22^ or BDDS Center.^23^ Certain platforms of this group focus on a specific disease, such as cBioPortal^24^ or G-DOC^25^ for cancer or COPD Knowledge Base^26^ for pulmonary dysfunction. The advantage of solutions based on existing computational infrastructure is direct use but at the cost of reduced flexibility in configuration and toolset management. The other group of solutions for translational medicine requires deployment on the user's infrastructure, often requiring substantial storage or high-performance computing (HPC) capabilities. Notable examples in this group are BRISK,^27^ tranSMART,^28^ and Transmed.^29^ Because of their highly configurable nature, such solutions are suitable in projects implicating sensitive data, and where a repository is needed to support ongoing projects, such as in case of longitudinal cohort^‡^ studies. Informative use cases of such platforms are SHRINE^30^ and DARiS,^31^ where well-defined demands of clinical research projects drove the design and implementation of infrastructure supporting translational medicine.

Visually aided data exploration is an important component of clinical and omics integration platforms. A notable contributor in this field is the Informatics for Integrating Biology and the Bedside project (i2b2, www.i2b2.org), a scalable framework enabling the use of clinical data for discovery research.^32,33^ The i2b2 Hive^34^ is a powerful collection of interoperable tools ranging from repository services to basic data conversions provided by i2b2 cells. Importantly, i2b2 Hive does not support directly the analysis of omics data, such as gene expression or whole-genome sequences by itself,^35^ but enables key capabilities of clinical data exploration and processing to be used by other platforms.

### Bioinformatics workflow management systems

Reusable and interoperable bioinformatics workflows become increasingly important in reproducible analysis of biomedical data and metadata, including clinical, omics, imaging, and sensor data.^36–38^ A number of software frameworks were developed to support the scientific community in this goal. In a thorough review and classification of these workflow frameworks, Leipzig^36^ groups existing technologies according to their interaction mode into command-line/application programming interface (API) and workbench approaches. The first group includes Snakemake,^39^ Yabi,^40^ Chipster,^41^ or Mobyle^42^ and relies on textual workflow construction in a script-like format. Certain tools in this group, such as Chipster, enable Web-based collaborative development of workflows.

The second group of platforms provides the so-called “workbench environment”: a GUI enabling visually supported construction of workflows. Usually, workflows are represented as graphs, where nodes correspond to data processing steps, and edges to data flow. Workbench solutions include Galaxy,^43^ Taverna,^44^ Pipeline Pilot,^45^ KNIME,^46^ or gUSE.^47^ Similar to data integration platforms, these tools need to be deployed on the user-provided infrastructure, and the extent of possible analysis is restrained by available storage and HPC capacities.

Ensuring computational resources may be a challenging task, and cloud computing becomes increasingly more important paradigm in development and execution of bioinformatics workflows. Cloud-oriented workflow management systems offer API support for construction of an analytical pipeline, including open-access solutions, such as Agave^48^ or Arvados,^49^ or a number of commercial services.^5^ Workbench platforms are also available in the computational cloud environment. Interestingly, a number of open-access solutions use Galaxy as a workflow construction engine, including Galaxy Cloud,^50^ Tavaxy,^51^ or Genomics Virtual Laboratory.^38^ Commercial cloud workbenches, such as Seven Bridges (http://sbgenomics.com), are also available. In summary, cloud computing is an attractive scalable option on demand, especially for multisite collaborative research projects in terms of bringing the tools to the data. However, the speed of data transfer to the cloud, flexibility of the configuration of analytical pipelines, and the issues of privacy and security in data analytics remain challenges to address.^15,36^

### Platforms for visualization of molecular interaction networks

With the progress of systems biomedicine, molecular interaction networks^§^ became a popular form of representing knowledge about molecular mechanisms pertinent to human health.^52^ First, such networks provide a necessary format to encode multitude of interactions identified in biomedicine. Second, they provide a good support for visual exploration and analytics of complex knowledge.^53^ As such, they have a great potential in aiding the interpretation of analytical outcomes of translational medicine pipelines.

Molecular interaction networks can be constructed in various ways that determine their size and purpose. Experiment-derived networks are established from different types of molecular readouts, allowing, with a certain probability, ascertain physical interaction between molecules, for example, protein–protein interaction^54^ or chromatin immunoprecipitation assays.^55^ Analysis-inferred networks are constructed by analyzing high-throughput omics data to identify molecules with similar properties or behavior, for example, using coexpression analysis.^56^ Finally, knowledge-based networks are established on the basis of existing body of knowledge, usually a set of published articles. Construction of knowledge-based networks is usually accomplished with text mining approaches^57^ or expert curation.^58,59^

While experiment-derived and analysis-inferred networks offer a vast amount of unbiased information, they are usually large-scale graphs, requiring sophisticated network analysis to draw meaningful conclusions. Mapping translational medicine data sets on top of these networks may be considered an important step in the analysis^60^ but not in the final interpretation of an analytical workflow. In turn, knowledge-based networks are usually established on the basis of low-throughput, in-depth experiments and allow for direct data interpretation. In particular, text mining networks are often used by the scientific community, where a number of commercial solutions, such as Ingenuity Pathway Analysis,^61^ Pathway Studio,^62^ or MetaCore,^63^ offer already established databases. These solutions, however, tend to contain the entire discovery pipeline inside their platforms, greatly reducing data interoperability.

Expert-curated networks are focused resources of high-quality confirmed knowledge and offer the highest degree of data set interpretation to translational medicine researchers. Important resources in the field of expert-curated networks are repositories called “pathway databases,” such as KEGG,^64^ Reactome,^65^ or WikiPathways,^59^ which describe general biomolecular mechanisms. In contrast, the other type of networks focuses on representing mechanisms of human diseases as so-called “disease maps.”^58,66,67^ Detailed representation of domain knowledge and support by domain-related literature makes disease maps a potentially interesting element of translational medicine analytical pipelines. Computational architectures supporting these maps provide dedicated APIs,^68,69^ opening an interesting avenue in translational medicine data processing—from storage, through bioinformatics workflow analytics, to interpretation by visualization on the dedicated molecular interaction network.

## Approach

A flexible workflow for translational medicine big data needs to provide biomedical experts, such as medical doctors and life scientists, with a possibility to explore high-dimensional data sets. Given the complexity of source data, experts need to be able to flexibly define constraints and filters to focus on the most representative data points for particular health-related questions. Selected data points need to be processed, often in multiple analytical steps, as biomedical data are heterogeneous and represent complex readouts. Finally, biomedical experts need to interpret their findings in the context of biological mechanisms to formulate hypotheses on disease mechanisms.

We decided to focus on translational medicine workflow providing the possibility of visually aided data exploration and informative hypothesis formulation. Therefore, our data integration platform of choice was tranSMART as it is a server-based solution with i2b2 data exploration component. We chose Galaxy as a workflow management system, considering its flexibility and the availability of tools. Finally, to provide informative interpretation of analytical results, we bridged the Galaxy Server with MINERVA platform, allowing overlay of exported data on disease-related mechanisms.

We approached this problem in three steps:

1. Data integration and exploration are handled using tranSMART repository^28^
2. Analysis of tranSMART-provided data is supported by Galaxy Server workflows^43,70,71^
3. Visualization of Galaxy-provided results is enabled via domain-specific knowledge repositories.^58^

**FIG. 1.**
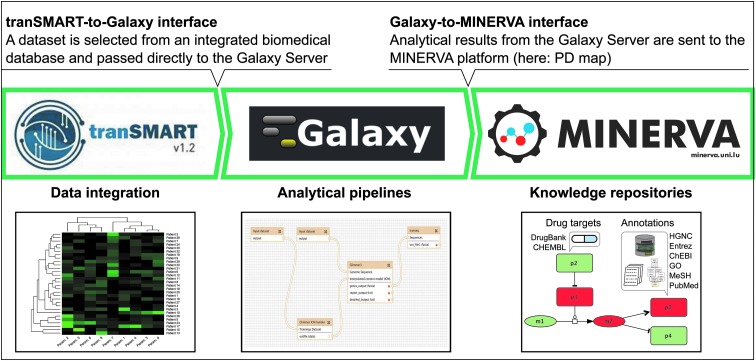
A workflow for big data analytics in translational medicine. Clinical and “omics” data are integrated in the tranSMART database, allowing their exploration and selection of relevant subsets for downstream analysis. Selected data set is automatically transferred to Galaxy Server as a source for user-defined analytical pipelines. Finally, the results of the analysis are automatically transferred to an associated knowledge repository hosted on MINERVA platform (here: PD map) and displayed on the visualized molecular interaction networks. PD, Parkinson's disease.

The workflow, as illustrated in detail in Figure 1, assumes a biomedical expert supervising each of the steps, while dedicated interfaces support automated data transition between each step.

### Integration and exploration of clinical and molecular data in tranSMART database

Translational medicine data sources are heterogeneous and of various granularities,^2,72^ and visually aided data exploration^73^ is an important enabling technology for biomedical experts. The powerful visualization and interoperability functionalities of i2b2 are coupled together with omics integration in tranSMART^28^ platform. tranSMART is a well-established platform enabling translation of preclinical research data into meaningful biological knowledge.^74^ It supports integration of low-dimensional clinical data and high-dimensional molecular data in a data warehouse architecture. Although tranSMART by default relies on a relational database technology, it extends toward storing the high-dimensional biological data using NoSQL technology HBase.^75^

The platform features data interoperability connectors, including clinical information collection,^76^ imaging data,^77^ visual analytics,^78^ or bioinformatics workflow management.^79^ Finally, tranSMART features built-in data mining and analysis applications based on open-source systems, such as i2b2 and GenePattern,^28^ and provides plugins to external tools, such as Dalliance Genome Browser,^80^ or APIs for statistical packages, such as R.^81^

For the abovementioned reasons, tranSMART became a technology of choice for European Translational Information and Knowledge Management Services (eTRIKS, www.etriks.org) initiative. eTRIKS provides infrastructure for data staging, exploration, and use in translational research supported by Innovative Medicines Initiative (IMI). IMI is a collaborative scheme, in which academic institutions and pharmaceutical companies in Europe conduct large-scale biomedical research.

To take advantage of the multiple functionalities of tranSMART, the target data sets have to be integrated following strict rules of data harmonization, semantic alignment, and quality checking. The data sets are curated following three common steps:

1. Data extraction: Source raw data files are extracted from either public or private data repositories. This could be a simple FTP transference from a Web repository or a database dump from a database management system, such as MySQL or Oracle™.
2. Data retrieval: Target information from the raw source files is identified and converted as Standard Format Files as defined by tranSMART curation guidelines. At this step, subject-level to sample-level data mapping is established.
3. Data annotation: Completing and standardizing annotations of metadata are also expected for guaranteeing data provenance.

**FIG. 2.**
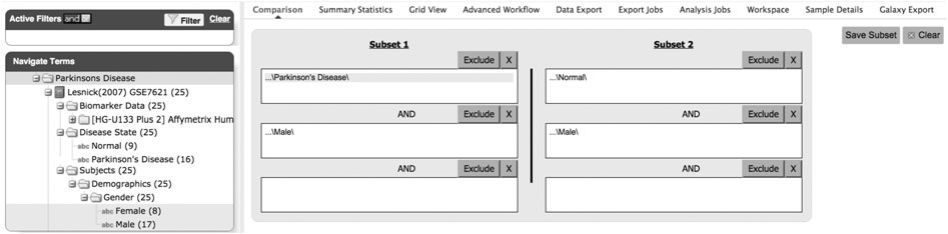
Cohort/subset definition based on the variables displayed in data tree. Two distinct subsets are defined based on the variables “disease state” and “gender.” In the left panel: data tree in tranSMART data set explorer. The data tree for GEO study GSE7621 following curation and loading to tranSMART is shown here. The data leafs correspond to the low- and high-dimensional data variable names. GEO, Gene Expression Omnibus.

The final product of the abovementioned steps is a set of Standard Format Files, which are used as input by tranSMART's native ETL (Extract, Transform, and Load) scripts. After data curation and loading to tranSMART, features collected for subject and samples become variables of the corresponding data set. These variables, as well as the relationships among them, are represented as a hierarchical parent–child tree control structure (or simply “tree,” see Fig. 2). This tree can be gradually expanded, which allows efficient data sets exploration and also the selection of variables from the hierarchy to build customized patient subsets for downstream analysis. Features that characterize desired data points in the tree, such as “age,” “gender,” or “disease state,” could be used as filters to narrow down the selected group. With tranSMART, researchers can pinpoint groups of patients and samples sharing similar characteristics, allowing straightforward hypothesis formulation. Easy identification of such coherent groups is a necessary prerequisite for accurate downstream analysis.

tranSMART platform has certain limitations concerning the size of handled data sets. First, data curation and integration are very time-consuming steps, which are necessary to upload a multidimensional data collection to tranSMART. Only then, users benefit the most from visually aided data exploration and interpretation. Second, considering the growing volume of data collected per patient,^12,15^ data storage may become a bottleneck in the proposed architecture. In our experience with tranSMART, even when working with a data set of 15,000 patients each with 2000 clinical variables, the system was responsive (data not shown). However, storing omics for these patients in the native tranSMART database is an issue. NoSQL solutions can be considered to address the problem of both storage and analysis of large data.^75^ Final bottleneck we foresee concerns the visualization capabilities of tranSMART. Displaying large amounts of data points via Web browsers is inefficient and may become a burden for large data sets.

### Analysis of selected data points using Galaxy Server

The process of selection and filtering of tranSMART data results in a focused subset, which is suitable to answer a particular research question of a biomedical expert. For this to happen, an analytical workflow needs to be designed, pinpointing key characteristics of the selected subset.

Galaxy as a bioinformatics workflow management system is available as both Web server and cloud workbench, offering flexibility in terms of data interoperability and allocation of computational resources.^43,50,51^ The Galaxy environment automatically and transparently tracks every detail of the analysis, allows the construction of complex workflows, and permits the results to be documented, shared, and published with complete provenance, guaranteeing transparency and reproducibility.^50^ Galaxy Tool Shed^82^ is a repository of more than 3000 community-developed tools, allowing easy and versatile establishing of bioinformatics workflows.^70,71^ Such workflows may combine different aspects of expert knowledge required in subsequent analytical steps. Basic knowledge about the system is sufficient to use default elements in the workflow construction. These default methods can be modified, where the user has sufficient expertise. Once the workflow step is done, users can easily share and modify it. Analytical results can be directly visualized using embedded functionalities or exported for downstream processing.

Data interoperability and flexibility are important features of Galaxy. The platform is available in both server and cloud-based versions and bridges to the other major bioinformatics workflow management systems—Taverna,^51^ KNIME, and gUSE.^37^ Such architecture permits transparent and replicable design of analytical workflows for data exploration and formulation of data-driven hypotheses.

Galaxy may face similar data volume-related issues as discussed above on tranSMART. In case of big omics data sets, data transfer and analysis may become time consuming, especially for large subsets chosen for analysis and complicated workflows. A possible solution to consider in such a case are advanced computational architectures offered by other workflow managers, such as gUSE. This solution is feasible to consider in the light of recent results on KNIME–Galaxy–gUSE workflow translation.^37^

### Interpretation of analytical results using contextualized knowledge repositories

High-dimensional translational medicine data sets are difficult material to draw conclusions relevant for human health. Data sets exported preselected from tranSMART database and analyzed using Galaxy will either, in many cases, remain multidimensional data sets or will be reduced to the list of prioritized molecules. Interpretation of such results remains challenging and requires both contextualization and visualization. Galaxy Server allows various export options. As the last step of our pipeline, we propose to interpret the results of analysis of Galaxy Server in the context of dedicated knowledge repositories supported by MINERVA platform, such as Parkinson's disease (PD) map.^58,69^ In particular, molecules prioritized by the constructed pipeline are automatically visualized on molecular interaction networks hosted by MINERVA platform.^69^

Knowledge on detailed molecular mechanisms can be assembled in the context of a given biological mechanism or a particular perturbation of this mechanism—a disease. Among others, Systems Biology Graphical Notation (SBGN)^83^ is used as a format for such mechanistic descriptions. Importantly, SBGN foresees a diagrammatical description of molecular mechanisms, introducing an important aspect of visualization to their curation. In effect, a “map” of molecular processes can be drawn and then visually explored for a comprehensive understanding of complex interactions. A number of systems biology-oriented maps were established following this paradigm.^84–86^ More importantly, the so-called “disease maps” gained interest as a way to assemble an overview of pathways and perturbations specific to a given pathology.^58,67,87,88^

MINERVA platform is a Web server supporting curation and visualization of SBGN-compliant molecular interaction networks. The maps of biological processes can be drawn in editors supporting SBGN notation, such as CellDesigner (www.celldesigner.org) or SBGN-ED (www.sbgn-ed.org), and uploaded to an instance of MINERVA Web server. There, the maps are automatically verified and annotated and become accessible for exploration via Web browser. MINERVA features dedicated functionalities coupled with Google Maps API to enable intuitive visual exploration, interaction with visualized content, advanced search queries, and mapping experimental data on the displayed networks. In turn, drug-targeting interface facilitates health-related interpretation or hypothesis generation.

## Results

We have combined three server-based platforms addressing different aspects of data processing in translational medicine research—data integration and exploration, bioinformatics workflow construction, and interpretation of analysis results in the disease context. In our choice of technologies, we focused on two criteria—capability for exploratory hypothesis generation and data interoperability. The platforms of our choice, tranSMART, Galaxy, and MINERVA, can be deployed as a single data processing workflow for translational medicine.

We focused on available PD studies and exercised our workflow as described above, from data set selection and filtering in tranSMART, through analysis in Galaxy Server, to interpretation of results in the PD map—an open-access dedicated knowledge repository. We have established a dedicated Virtual Machine** to demonstrate the functioning of our workflow. To provide data sets for exploration, we have used tranSMART PD data sets we previously curated, which are also available at https://public.etriks.org.

### Integration and visual exploration of PD data sets in tranSMART

For the first step of our workflow, we used PD-related studies that are publicly available in the Gene Expression Omnibus (GEO) database.^89^ To integrate the GEO studies, data curation was performed to meet the required format of tranSMART,^74^ as discussed above. In this use case, we worked with the GSE7621 PD study data^90^ for defining two focused cohorts using tranSMART data set explorer.

Study-related variables in tranSMART can be assigned to two broad categories: low- and high-dimensional data. Low-dimensional data correspond mostly to clinical, patient-centric data (e.g., systolic blood pressure) and low-throughput diagnostic measurements (e.g., quantification of a disease-related blood biomarker). The corresponding values of low-dimensional data are usually available as text or numeric values. High-dimensional data, in the majority reflecting “omics” data, are structured as a numeric matrix.

For the purpose of this work, we used tranSMART for defining two specific patient cohorts based on low-dimensional data. We used tranSMART data set explorer to traverse the data tree displaying the low- and high-dimensional data variables for a given study (Fig. 2). Using associated drag-and-drop functionality, we performed on-the-fly cohort definition. As can be seen in Figure 2, the two cohorts have been defined based on the variables “disease state” and “gender.” Having these two cohorts, we were in subsequent steps to export their high-throughput data sets containing gene expression profiles of the patient brain samples for downstream analysis and visual exploration.

### Interface: tranSMART to Galaxy Server

Once subcohorts are built using the i2b2 tree, all data related to the two subcohorts can be exported as tab-delimited files. This step is possible as tranSMART data interface enables export of all selected data to be shared with analytical tools. To make the gene expression data available to the Galaxy environment for further analysis, we used tranSMART data export functionality. This connection has been implemented within the collaboration of the eTRIKS consortium and the tranSMART foundation. In particular, exported data can be streamlined automatically to an associated Galaxy Server via the Galaxy plugin to tranSMART (https://github.com/thehyve/transmart-galaxy).

The tranSMART–Galaxy interface uses the export function of tranSMART and transfers the files via Galaxy API to the Galaxy Server. User of Galaxy will then have access to the exported data of the subcohorts built in tranSMART. This way, preselected microarray data then become available in the Galaxy Server Workspace (GSW) for further analysis.

Both tranSMART and Galaxy provide user access rights management functions. Here, we rely on security mechanisms natively provided by the two systems. The interface requires a preconfigured login–password pair to upload data to a dedicated GSW. The login–password pair is then used as a parameter in the interface configuration, such that only users having access rights to both systems can establish the interface and execute the data transfer over it.

### Analysis of a selected subset on Galaxy

High-throughput data provided by tranSMART contain gene expression in samples from the two selected cohorts: males with PD (four samples) and age-matched healthy males (eight samples). The data files are automatically available in GSW after their export from tranSMART and can be used as input files.

**FIG. 3.**
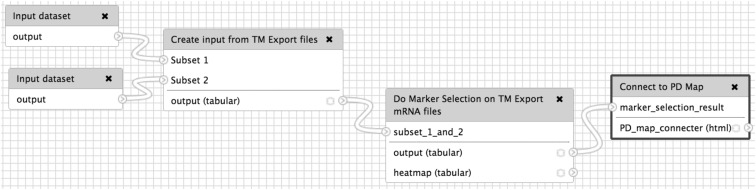
Visually constructed data flow in the Galaxy Server comparing two cohorts from tranSMART.

We have designed a dedicated Galaxy workflow (Fig. 3). The workflow is subdivided into steps from incorporation of the input files taken from tranSMART through performing the differential expression analysis and uploading the obtained results to the PD map hosted on the MINERVA platform and making them accessible for interpretation in the disease-specific context.

A comparison between these two data sets provides insight about disease-related mechanisms that may be cohort specific. This differential gene expression was calculated as predefined method using Bioconductor package “limma” in Galaxy^91,92^ (absolute fold change >1.5, *p*-value <0.05). The resulting list of 3286 differentially regulated genes was uploaded via MINERVA to the PD map for visual interpretation. This process led to the labeling of 224 different genes and/or their related protein products in the PD map.

### Interface: Galaxy Server to MINERVA

MINERVA platform accepts POST requests, where the user specifies the target molecular network, user, password, and the data set to be uploaded. To ensure seamless data transfer from Galaxy to MINERVA, we created a step in the GSW called “PD map connector.”^††^ This step generates a POST request to the associated MINERVA instance—PD map in this case—to generate a custom visualization on the basis of the workflow data.

In the backend of the target MINERVA instance, a temporary session will be created for that particular data set to generate a custom layout, which will be available in the “Layouts” tab after user logs in. The uploaded data set may contain different types of elements, allowing coloring elements corresponding to multiple “omics” or interactions of the visualized network.^69^

By seamlessly connecting Galaxy Server to MINERVA platform, the users can securely transfer analysis results obtained from Galaxy workflows to MINERVA platform without leaving the Galaxy system. As shown below, visualization of the results on the PD map allows the identification of major molecular pathways perturbed in postmortem brain tissue of male Parkinson's patients, as selected in tranSMART and processed in Galaxy.

### Upload and interpretation of analysis results in the PD map

The data exploration and analysis steps described above created a list of molecules characterizing the PD-related cohort in comparison to the controls. This list is then projected on the PD map, a contextualized knowledge repository of mechanisms relevant for the disease. The repository is hosted using the MINERVA platform, a Web service for visualization of molecular networks, with the capability of custom data upload and mapping.^69^ Pathways and processes displayed in the PD map provide disease- and cellular context-related information.^93^ More than 1500 molecular interactions displayed in the PD map are from more than 1000 PD-related publications manually curated by experts.^58^

**FIG. 4.**
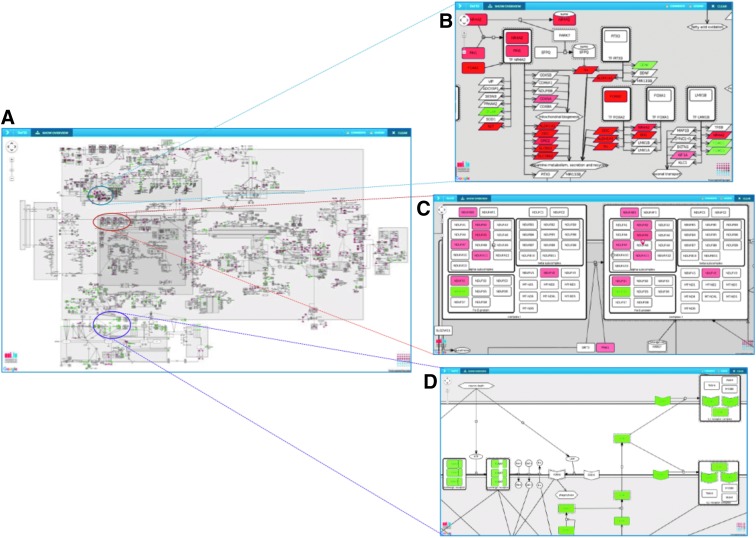
Data visualization and analysis using PD map. **(A)** Differential gene expression data comparing postmortem brain tissues from male PD patients versus controls are displayed on the PD map (green, upregulated; red, downregulated). Pathways and processes of conspicuous areas (colored circle) could be identified using the pathway and compartment layout view of the PD map. Detailed view on deregulated genes that encode for proteins involved in dopamine metabolism, secretion, and recycling **(B)**, on mitochondrial electron transport chain, in particular elements of complex I **(C)**, and on microglia activation **(D)**.

Evaluation of highlighted areas in the PD map shows pronounced alterations in the cell nucleus, in particular a battery of downregulated (red) genes involved in metabolism and secretion of the neurotransmitter dopamine (Fig. 4, blue circle^‡‡^).^94^ Another visible perturbation affects the mitochondria, in particular elements of complex I (Fig. 4, red circle^§§^). This process is essential for energy homeostasis, in particular in high energy demanding neurons. Finally, we observe upregulation (green) of processes involved in neuroinflammation (Fig. 4, purple circle***).^95^ On the basis of this visual exploration, data analyst may get comprehensive insights in molecular processes potentially involved in the disease of this specific patient cohort supporting new insights for diagnosis, prognosis, and therapy. Another approach for visualization is the drug target interface integrated in the MINERVA platform, enabling the mapping of potential drug interactions with elements of the map, suggesting more precise treatments and possibly an improvement in existing therapies.^96^

## Conclusions

Visualization is a necessary tool on the interface between the expert and big data processing pipelines. It is especially important in the field of translational medicine, where biomedical experts formulate and test their hypotheses about new diagnostic approaches or treatment. This process can be greatly supported with the available translational medicine big data, including clinical and molecular data sets.^97^ Efforts in this direction are reflected with development of disease-oriented knowledge repositories, for example, for pulmonary^26^ or neurodegenerative disorders.^98^ Nevertheless, these knowledge bases lack seamless data flow and require a number of explicit data transformation steps for exploratory analysis. In turn, less technically versed users are restrained in data-driven hypothesis generation.

Currently, a single person has to master a wide range of skills to perform a complete biomedical data analysis and interpretation. This is one of the reasons that big data integration, analytics, and interpretation become the true bottleneck of translational medicine.^15^ We address this issue by seamlessly combining platforms supporting these steps, each of them having strong components of visually aided data exploration and analysis. Our approach is modular and capitalizes on strong points of each of the platforms, promoting data interoperability. Similar efforts have already been proposed,^99^ involving tranSMART as data integration platform and a commercial solution GeneData as the analysis and interpretation engine. We believe that our pipeline extends their approach by involving a disease-related knowledge repository and by involving only open-access technologies will be useful for the scientific community.

The platforms of our choice are server based, allowing construction of the entire pipeline in a protected environment, avoiding ethical and legal issues present in the cloud scenarios. Nevertheless, cloud computing paradigm is compelling, especially for researchers having limited storage and HPC capabilities.^5,16^ Efforts in this direction are promising^31^ and need to be supported by further advances in data interoperability.^12^ We believe our work is a step in this direction.

## Notes

^*^ **Clinical data:** Data collected by the characterization of a biomedical research participant by a medical professional, for example, demographics, study-specific questionnaires, or examinations. **Molecular data:** Data collected by analyzing samples donated by a biomedical research participant using imaging (microscopy) or high-throughput molecular profiling (“omics”).

^†^ **“omics”:** Technologies for characterization and quantification of entire pools of biological molecules in a given sample. Data sets generated using omics are highly dimensional, ranging from hundreds to hundreds of thousands of variables per sample. The name “omics” encompasses particular readout methods: genomics (entire genome), transcriptomics (entire gene expression profile), proteomics (entire protein expression profile), metabolomics (entire pool of metabolites), and others.

^‡^ **Cohort:** A group of people with a shared characteristic. Here, a group of subjects with demographic, clinical, or other characteristics relevant for translational research.

^§^ **Molecular interaction networks:** A class of graphs, where nodes represent various biomolecules, and edges represent interactions between them.

^**^ Available at http://r3lab.uni.lu/web/tgm-pipeline

^††^ Code available on the project website, http://r3lab.uni.lu/web/tgm-pipeline

^‡‡^ In the PD map: http://tgm-pipeline.uni.lu/minerva/?search=%22TF%20NR4A2%22

^§§^ In the PD map: http://tgm-pipeline.uni.lu/minerva/?search=reaction:re720

^***^ In the PD map: http://tgm-pipeline.uni.lu/minerva/?search=IL1%20receptor%20complex,purinergic%20receptor

## Abbreviations Used

API: application programming interface
ETL: Extract, Transform, and Load
eTRIKS: European Translational Information and Knowledge Management Services
GEO: Gene Expression Omnibus
GSW: Galaxy Server Workspace
HPC: high-performance computing
IMI: Innovative Medicines Initiative
PD: Parkinson's disease
SBGN: Systems Biology Graphical Notation
